# Voluntary undergraduate technical skills training course to prepare students for clerkship assignment: tutees’ and tutors’ perspectives

**DOI:** 10.1186/1472-6920-14-71

**Published:** 2014-04-04

**Authors:** Mats Blohm, Markus Krautter, Jan Lauter, Julia Huber, Peter Weyrich, Wolfgang Herzog, Jana Jünger, Christoph Nikendei

**Affiliations:** 1 Department for General Internal and Psychosomatic Medicine, University Hospital of Heidelberg, Heidelberg, Germany; 2 Department of Nephrology, University Hospital of Heidelberg, Heidelberg, Germany; 3 Department of Internal Medicine IV (Diabetes, Endocrinology, Nephrology and Clinical Chemistry), University Hospital of Tübingen, Tübingen, Germany; 4 Centre for Psychosocial Medicine, University of Heidelberg, University Hospital for General Internal and Psychosomatic Medicine, Thibautstrasse 2, Heidelberg 69115, Germany

**Keywords:** Medical education, Peer assisted learning, Clerkship preparation, Tutor training, Clinical skills

## Abstract

**Background:**

Skills lab training has become a widespread tool in medical education, and nowadays, skills labs are ubiquitous among medical faculties across the world. An increasingly prevalent didactic approach in skills lab teaching is peer-assisted learning (PAL), which has been shown to be not only effective, but can be considered to be on a par with faculty staff-led training. The aim of the study is to determine whether voluntary preclinical skills teaching by peer tutors is a feasible method for preparing medical students for effective workplace learning in clerkships and to investigate both tutees’ and tutors’ attitudes towards such an intervention.

**Methods:**

A voluntary clerkship preparation skills course was designed and delivered. N = 135 pre-clinical medical students visited the training sessions. N = 10 tutors were trained as skills-lab peer tutors. Voluntary clerkship preparation skills courses as well as tutor training were evaluated by acceptance ratings and pre-post self-assessment ratings. Furthermore, qualitative analyses of skills lab tutors’ attitudes towards the course were conducted following principles of grounded theory.

**Results:**

Results show that a voluntary clerkship preparation skills course is in high demand, is highly accepted and leads to significant changes in self-assessment ratings. Regarding qualitative analysis of tutor statements, clerkship preparation skills courses were considered to be a helpful and necessary asset to preclinical medical education, which benefits from the tutors’ own clerkship experiences and a high standardization of training. Tutor training is also highly accepted and regarded as an indispensable tool for peer tutors.

**Conclusions:**

Our study shows that the demand for voluntary competence-oriented clerkship preparation is high, and a peer tutor-led skills course as well as tutor training is well accepted. The focused didactic approach for tutor training is perceived to be effective in preparing tutors for their teaching activity in this context. A prospective study design would be needed to substantiate the results objectively and confirm the effectiveness.

## Background

Skills lab training has become a widespread tool in medical education, and nowadays, skills labs are ubiquitous among medical faculties across the world. The opportunity to practice both technical medical procedures and communication skills by means of mannequins, standardized patients and role-play makes a valuable contribution to medical students’ professional development. Therefore, structured clinical training in the sheltered learning environment of skills labs has become a vital part of modern academic medical education, allowing efficient preparation for future patient encounters. Over the past years, evidence has been gathered which supports the effectiveness of skills lab teaching with respect to patient safety, the physician-patient relationship and performance in clinical exams [1-4].

An increasingly prevalent didactic approach in skills lab teaching is peer-assisted learning (PAL), which implies the assignment of either advanced medical students as peer tutors for lower-level fellow students (cross-year teaching) or tutors teaching same-year fellow students (same-year PAL) [5]. It has been shown that peer teaching in skills labs is not only effective, but can be considered to be on a par with faculty staff-led training [6-8]. Furthermore, acceptance of peer teaching is extraordinarily high among tutees, and the competence and patience of well-trained peer tutors is widely appreciated [9]. In addition to the effectiveness in achieving tutees’ learning goals, even the advanced students volunteering as tutors benefit from their work [7,10], predominantly through the development of their own clinical and teaching skills and from the positive feedback obtained by their tutees, thus creating a highly pleasant learning atmosphere and a win-win situation for all [9]. However, thorough and structured didactic and technical training of the tutors prior to their teaching activity appears to be crucial [11], although further research on this aspect is required.

Offering the opportunity to explore the future workplace and to gather clinical experience by learning to care for patients in a responsible manner, medical clerkships represent a pivotal part of medical education. During their clerkships, medical students have the opportunity to apply their clinical-technical and communication skills in interaction both with an inter-professional team of medical staff and with real patients. Thus, they provide a unique way to apply the knowledge gathered during the academic curriculum and transfer it to real-life situations. This type of workplace learning may be a milestone on the path to future work as a physician. However, a rewarding and motivating workplace experience requires an active integration and good interaction between the students, medical staff and patients, which can be ensured by appropriate preparation and training [12]. It has long been known that clerkships alone are not sufficient for the acquisition of adequate practical skills [13], but an integrated skills training combining structured, longitudinal curricular skills training with clerkship experience is assumed to be more effective in preparing students for real-life situations [12]. This is also reflected in better OSCE results of students who have received integrated skills lab training compared to those receiving traditional bedside teaching alone [14,15]. However, while a limited number of studies have focused on the concept of peer-assisted learning in the context of mandatory curricular skills lab training during clinical education [9], to our knowledge, no studies have been published assessing the acceptance of peer teaching, tutor training and attitudes in a voluntary skills lab training setting designed as a structured clinical preparatory course for clerkships of preclinical medical students.

The aim of the study is to determine whether voluntary preclinical skills teaching by peer tutors is a feasible method for preparing medical students for effective workplace learning in clerkships and to investigate both tutees’ and tutors’ attitudes towards such a course. To this end, the research questions were (1) whether participating tutees perceive personal progress, (2) whether they profit from tutors’ teaching competency, and (3) whether they perceive an increase in self-assessment of procedural skills in pre- and post-evaluation forms. Furthermore, tutors were interviewed to identify crucial aspects for successful teaching and adequate tutor training. Here, the research questions were to assess (4) their attitudes, concerns, feedback and suggestions in an exploratory manner, and (5) whether the tutor training is well accepted by tutors.

## Methods

### Definition of learning objectives

First of all, suitable learning objectives for the clerkship preparatory course were defined. As the course was supposed to equip mainly first- and second-year students with clinical skills beneficial for their first medical clerkship, a list of basic and essential clinical technical skills was compiled on the basis of literature research [16] and expert discussion (n = 4). Ultimately, eight procedural skills were identified as the most important, as listed in Table 1. It was decided that basic communication should be taught alongside the technical procedures, as called for in the literature [17,18].

**Table 1:** Procedural Skills as taught in the peer-assisted voluntary clerkship preparation skills course

**Overview of skills lab training sessions**^**a**^	
**Training session 1**	**Training session 2**
Blood pressure measurement	Schellong Test
Blood sampling	ECG placement
Peripheral venous cannulation	Basic surgical work routine
Subcutaneous and intramuscular injection	Simple interrupted surgical suture

^a^Each training session was carried out with a maximum tutor: tutee ratio of 1: 5.

### Design of the voluntary clerkship preparation skills course

An approach with the course content distributed across two afternoons (one week apart) was chosen. Each session lasted for two and a half hours and comprised the practicing of four technical medical procedures, with approximately 30 minutes designated per skill. Tutees practiced the procedural technical skills either on each other (blood pressure measurement and Schellong test) or on mannequins (all other skills). Communication was integrated into the teaching of every practical skill through the provision of specific suggestions on how to implement patient-centered communication and guide the patient through the procedure. In addition, the skill stations were designed in the form of a role-play in order to deliberately focus on patient communication [18]. In the course manual (see below), basic rules for feedback in the skills lab setting were described in order to foster awareness of the importance of feedback among the course participants. In this line, participants should be encouraged to ask for differentiated feedback from their tutor and to also apply feedback techniques on each other during the course.

The maximum number of participants was limited to 20. This resulted in four groups of up to five students rotating between the four skill stations (see Table 1). Each station was led by a peer tutor. The short theoretical introduction to each skill, providing background knowledge on, for example, indications for the procedure, was followed by a step-by-step demonstration by the tutor adhering to the previous training with the consultants. Tutees were then given the opportunity to practice the skill while receiving continuous feedback by their tutor. After the second course session was completed, participants were awarded a certificate stating the course content and duration, which was to serve as proof of qualification when applying for a clerkship.

### Peer tutors and tutor training

The clerkship preparation skills course was independently led by peer tutors (n = 10) who were part of the interdisciplinary longitudinal skills lab team [19]. Peer tutors were recruited on a voluntary basis from among the third- to fifth-year students of the medical school and already had teaching experience from curricular skills lab courses. The tutors were provided with financial compensation from the medical faculty by receiving contracts as student assistants.

Tutor training comprised three 3-hour sessions and was led by two consultants of internal medicine and a psychologist, thus ensuring appropriate technical and didactic training. With respect to technical skills, each procedure was demonstrated in a step-by-step manner by one of the consultants of internal medicine, who emphasized crucial aspects of the procedure and potential pitfalls. Tutors (n = 10) then repeatedly practiced the skill under close supervision of the consultant, receiving immediate feedback from both fellow tutors and the consultant. As a reference source, the tutors received a comprehensive training manual containing detailed checklists for every procedure taught during the training. The checklists were adapted from the catalogues used at the University of Heidelberg for feedback of role-plays in skills labs [18]. In addition to the practicing of all technical medical procedures, during the final and didactically-focused session, an introduction to structured and motivating feedback techniques [20] and role-play [18,21] was given, complemented by selected didactic tools, including Peyton’s Four-Step Approach [22]. By means of this standardized tutor training, consensus was achieved on how each skill should be taught and the different levels of experience among the tutors converged with each other, thus ensuring standardized teaching during the clerkship preparation skills course.

### Tutees

Students at the medical faculty at the University of Heidelberg were free to enroll in the course via a web-based e-learning platform. Predominantly, students from the two preclinical years were invited to enroll, but it also appeared that even third-year students were interested in the course. Between November 2012 and July 2013, a total of 135 students decided to participate in the clerkship preparation skills course.

### Evaluation of peer tutor training

Each of the three tutor training sessions was evaluated by questionnaires provided before and after the training and comprised self-assessment with respect to the skills taught during the training and overall acceptance of the training. Self-assessment for technical skills was carried out by means of a positively worded 6-point Likert scale (1 = I fully agree, 6 = I strongly disagree) for each skill on the following aspects: a) knowledge of the individual steps of the procedure, b) ability to perform the procedure on a mannequin, c) ability to perform the procedure on a patient, and d) ability to instruct a fellow student to perform the procedure. Using the same Likert scale, role-play training was assessed as follows: a) knowledge of the most important rules in role-play in a skills lab setting, b) ability to participate in role-play in an authentic way, c) ability to communicate with a patient in a patient-centered way during a procedure, and d) ability to instruct a fellow student during role-play. Feedback training was evaluated by means of the categories: a) knowledge of the most important feedback rules in skills lab training, and b) ability to give appropriate feedback to fellow students during skills lab training.

In addition to the evaluation of the individual skills, the post-evaluation involved an overall assessment of each training session. Fulfillment of expectations of the training was assessed by a verbal rating scale: (a) exceeding expectations, b) fully meeting expectations, c) partially meeting expectations and d) not meeting expectations). Standards of the training were evaluated as a) fully appropriate, b) mostly appropriate, c) partially appropriate, or d) not appropriate. Statements evaluated using a 6-point Likert scale (1 = I fully agree, 6 = I strongly disagree) are shown in Table 2. Finally, tutors were asked to give the training an overall grade according to the German school grading system (1 = excellent, 6 = failed).

**Table 2:** Pre-post self-assessment ratings and overall acceptance by trainees (n = 95)

**Item**^**a**^		**Pre-training**		**Post-training**				
	**N**	**Mean**	**SD**	**Mean**	**SD**	**T**	**df**	**p**^**b**^
**Intramuscular injection**	92	5.01	1.45	2.22	1.23	17.59	91	<.001
**Subcutaneous injection**	92	4.51	1.76	1.76	1.18	15.39	91	<.001
**Blood sampling**	92	2.66	1.13	1.89	1.02	5.32	91	<.001
**Peripheral vein cannulation**	92	5.05	1.22	2.68	1.12	17.17	91	<.001
**Blood pressure measuring**	59	1.78	1.07	1.47	1.19	1.64	58	.054
**Schellong test**	82	5.23	1.31	1.31	0.75	22.43	81	<.001
**ECG placement**	81	4.70	1.50	1.77	0.75	17.79	80	<.001
**Surgical suture**	78	5.50	0.96	2.29	1.02	22.92	77	<.001
**Role play**								
I am able to communicate with a patient during a procedure in a patient-centered way	80	3.43	1.31	2.40	1.35	7.15	79	<.001
**Basic surgical work routine**								
I am able to perform the procedure in the operating theatre	81	4.11	1.63	1.36	0.51	14.91	80	<.001
**Overall course acceptance**								
I feel well prepared for future clerkships	80			1.66	0.64			

^a^For the statement “I am able to perform the procedure on a patient” (where not otherwise specified)^a^ One-tailed tests; ^b^Results of Student’s t-tests for dependent samples.

Six-point Point-Likert-Scale (1 = I fully agree; 6 = I fully disagree).

### Evaluation of the peer tutor-led course by tutees

Course participants were supplied with evaluation questionnaires before and after both training sessions. The students were asked to provide self-assessment with respect to each of the technical skills. The assessment was aimed at measuring different levels of confidence regarding the performance of the respective procedure. Knowledge of the individual steps of the procedure, ability to perform the procedure on a mannequin, and ability to perform the procedure on a patient were assessed using a positively worded 6-point Likert scale (1 = I fully agree; 6 = I strongly disagree). With regard to accompanying communication skills, the following aspects were rated using the same scale: knowledge of the most important rules in role-play in a skills lab setting; ability to participate in role-play during skills lab training; ability to combine performance of the technical task with communication during role-play; and ability to communicate with a patient in a patient-centered way in a real-life situation. Comparison between self-assessment before and after the course allowed for the individual progress during the course to be evaluated.

Post-evaluation after the completion of the second session additionally contained an overall evaluation of the course. The following items were assessed: fulfillment of expectations for the course; feeling of being well prepared for medical clerkships; competence of peer tutors; confidence with respect to the management of difficult situations; and whether the tutee would recommend the course to fellow students.

### Evaluation of the peer tutor-led clerkship preparation skills course by tutors

In a parallel approach, peer tutors were asked for their thoughts and comments on the course by means of a questionnaire comprising four open-ended questions. Tutors were asked about their attitude towards a preclinical preparatory course for clerkships and to provide reasons based on their own experiences during clerkships and curricular education to support their point of view. Further questions assessed advantages and potential problems of a peer tutor-led approach. Finally, tutors were asked for suggestions and ideas for improving the course. Open-ended questions were: 1) Why do preclinical medical students need specific preparation for medical clerkships? 2) Do you think the implementation of a clerkship preparation skills course is helpful? 3) What advantages and disadvantages of a peer tutor-led approach can you find? 4) Do you have suggestions for improving the clerkship preparation skills course?

### Skills lab education at the University of Heidelberg

Skills lab teaching was established at our faculty in 2000 and has been continuously developed and extended ever since. All major clinical specialties offer skills lab teaching within the framework of the core curriculum, on either a mandatory or voluntary basis. Faculty staff-led teaching is complemented by peer-assisted learning, with the latter being voluntary in the majority of cases. A specific skills curriculum is offered for final-year students accompanying their internship.

### Statistical analysis

All data obtained from Likert scale ratings are shown as means ± standard error. Pre/post self-assessment comparisons were carried out using paired Student’s t-tests, and p-values of < 0.05 were considered as significant. The software package SPSS (version 22, 2013; IBM) was used for statistical analysis. After transcribing the audio files of the peer tutor interviews verbatim, a qualitative content analysis was performed following the principles of grounded theory [23]. First, we conducted an open coding of all of the interview transcriptions line by line. In detail, single or few sentences were identified as a code, representing the most elemental unit of meaning [23]. Next, the codes were summarised into relevant themes for each participant, using the software MaxQDA (2010 version, VERBI GmbH, Berlin). As themes were recurrent among different participants, themes were then compared and adapted, until relevant themes for all participants could be defined. The assignment of respective codes to specific themes was conducted by two independent analysers and subsequently discussed to reach consensus and, if required, adjusted. The analysers (one female, one male) both have long-standing expertise in qualitative research and are also certified skills lab trainers. In a final step, themes were consolidated into four relevant categories.

### Ethics

The ultimate goal of the study was curriculum improvement. Consequently, the ethics committee of the University of Heidelberg waived the requirement for an ethical approval procedure for above described study design. The study was conducted in accordance to the declaration of Helsinki, revised form, Seoul 2008. All participants gave written informed consent. Study participation was voluntary.

## Results

### Peer tutor sample

All tutors leading the voluntary clerkship preparation skills course (n = 10) were from the faculty’s PAL tutor pool and were in the third to fifth year of medical school (9 female, 1 male, resembling the female: male tutor ratio of our PAL tutor pool, mean age 23.9 ± 1.39 years).

### Student sample

135 (55 male, 80 female, mean age 22.2 years) students participated in the voluntary clerkship preparation skills course during nine course cycles between November 2012 and July 2013. The majority of the participants were from the first and second years of medical school (n = 127) and thus preclinical students. However, even a smaller number (n = 8) of third-year students who had just completed the preclinical section also volunteered to participate.

### Peer tutors’ evaluation of the tutor training

n = 10 tutors from the medical faculty’s PAL tutor pool attended tutor training. Eight tutors attended all three training sessions, while n = 2 only attended the second and third sessions, and n = 1 tutor missed the third session. Evaluation forms were obtained from n = 8 tutors for session 1, n = 10 for session 2 and n = 9 for session 3. Pre-post self-assessment rating and acceptance is depicted in Table 3 for all three training sessions.

**Table 3:** Pre-post self-assessment ratings provided by trainees (n = 10)

**Item**^**a**^		**Pre-training**		**Post-training**				
	**n**	**Mean**	**SD**	**Mean**	**SD**	**T**	**df**	**p**^**b**^
**Peripheral intravenous cannulation**	7	3.29	1.60	1.71	1.11	5.28	6	<.001
**ECG placement**	7	3.57	2.37	1.57	1.13	2.45	6	<.025
**Blood pressure measurement**	7	1.71	1.11	1.14	0.38	1.92	6	<.052
**Nasogastric tube placement**	7	3.71	1.98	2.14	1.07	3.67	6	<.005
**Schellong test**	10	2.90	2.33	1.70	1.34	2.45	9	<.019
**Blood sampling**	10	1.60	0.52	1.00	0.00	3.67	9	<.003
**Intramuscular injection**	9	3.00	1.58	1.44	1.01	4.13	8	<.002
**Subcutaneous injection**	9	2.78	1.56	1.44	1.01	4.62	8	<.001
**Simple interrupted suture**	9	3.00	2.00	1.56	0.88	3.51	8	<.004
**Basic surgical work routine**	9	3.11	2.26	1.11	0.33	2.83	8	<.011
**Role play**	9	3.33	1.50	1.67	0.71	5.00	8	<.001
**Feedback**	9	2.89	1.36	1.78	1.09	3.59	8	<.004

^a^For the statement “I am able to instruct a fellow student” and “I am able to give appropriate feedback to a fellow student” for feedback skills; ^b^Results of Student’s t-tests for dependent samples.

Six-point Point-Likert scale (1 = I fully agree; 6 = I fully disagree).

### Trainees’ acceptance of the voluntary clerkship preparation skills course

A total of 135 students have participated in the voluntary clerkship preparation skills course so far. Evaluation data were retrieved from seven course cycles (first two course cycles were not evaluated) from 95 participants who completed questionnaires before and after both training sessions (100%). Results for pre- and post-self-assessment as well as overall course ratings are shown in Table 2.

### Tutors’ attitudes towards the voluntary clerkship preparation skills course

Questionnaires assessing tutors’ attitudes towards the voluntary clerkship preparation skills course were sent to all peer tutors who had taught the course after three course cycles (n = 7). Complete questionnaires were received from n = 6 tutors and were considered for qualitative analysis (see Table 4).

**Table 4:** Citations related to categories derived from qualitative analyses of tutor statements(N=6; T1 – T6)

	**Category 1: Preparation for the clerkship (30 statements)**
	**Theme “Subject-based preparation” (16 statements)**
•	“To be able to practice the practical skills in advance in a quiet environment.” (T5)
•	“If a medical clerk has already mastered certain skills, he can relieve the medical personnel of a lot of work, which is rewarded with more time for teaching and a higher motivation of the teachers; this in turn fosters the clerks’ motivation and confidence in their own abilities; good preparation therefore increases the likelihood of benefiting from the clerkship.” (T4)
•	“In the hospital, often nobody has any time to show you something, but if you have already had practice in some things and you say that, then the physicians let you perform measures earlier, under observation, and this saves the physicians’ time, and on occasion you are asked to do certain things. Also, I find that you are not so overexerted because you’ve already been able to practice the different skills, and then in the practicum/clerkship, you can concentrate on the new things you are learning.” (T3)
•	“Through our preparation course, the students are already able to learn practical skills before their practicum. They can learn this in what I believe to be a very pleasant environment (namely from us student tutors), and at the same time also make mistakes without ‘looking stupid’.” (T6)
•	“Preparation imparts the necessary background knowledge behind the clinical skills. Why am I doing what I am currently doing? Why won’t something else work as an alternative? E.g. Stitching instead of letting a wound heal by itself.” (T2)
•	“Yes, I find the course useful as you are taught important skills as compactly as possible, which might be useful in the clerkship. Such a course offers the possibility to learn skills in a protected environment (not directly on the patient in the clinic); by taking place at uni and being led by tutors, you don’t have the feeling you have to do it well or that you can’t ask any questions, in contrast to learning in the clinic. In such a course, you have time to perform things correctly and carefully and to ask questions. In the course, you get taught the skills according to a standardized schema. In the clinic, by contrast, every physician does things differently, some don’t pay attention to important things like wearing gloves or disinfecting.” (T6)
•	“Because through preparation, self-confidence is increased and disappointments due to poor guidance in the clerkship are prevented.” (T4)
•	“The most important skills for the clerkship are taken into account.” (T4)
	**Theme “Preparation for patient contact” (8 statements)**
•	“Often, you perform the clinical skills for the first time on the patent and under time pressure, which turns the patient into a ‘guinea pig’ and also doesn’t impart any feeling of confidence to the student. Practice also imparts a certain confidence to the patients. He feels he is in good hands in the care of a student, which increases the relationship of trust between the two parties.” (T5)
•	“Lastly, I find it particularly important so that you feel surer of yourself and can face the patient in a confident manner.” (T3)
•	“Because in this way, the students can deal with patients more appropriately and competently and so the patients’ appreciation for care by students increases in the long term.” (T4)
•	“You can practice the skills on mannequins and in this way don’t have to be scared of hurting the patient. Moreover, in our course, you practice communicating well with the patients. We try to show the students that you can both conduct a conversation and perform the skill quickly and correctly.” (T6)
	**Theme “Facilitation of the daily routine on ward” (6 statements)**
•	“Through such a preparation course, we try to take away some of the students’ fears regarding their practicum. On the first days of a practicum you are confronted with lots of new things anyway, so you’re happy if you have already, for example, practiced drawing blood and are allowed to do it right away and it works.” (T6)
•	“It spares embarrassing situations and takes away or at least reduces the nerves, which then again leads to more confidence.” (T3)
•	“So that you don’t go under in the daily routine on ward.” (T1)
•	“I find a preparation for practica and clerkships useful because you then find it easier to integrate yourself.” (T3)
•	“Also, you can relieve the sisters of some work, e.g. measuring blood pressure, taking ECG etc., which fosters the work climate and takes some burden off the sisters.” (T3)
	**Category 2: Leadership of the course by student tutors (19 statements)**
	**Theme “Fewer inhibitions towards student tutors” (6 statements)**
•	“I find it useful that the course is led by tutors, as it makes the atmosphere really relaxed. The students have the courage to ask questions, and also ask questions which they would not ask a lecturer/professor as they would find it awkward.” (T3)
•	“Also, the inhibition threshold towards other students is not so high, meaning that also in this environment, errors are allowed to take place which can be calmly corrected – without the student possibly feeling labeled as ‘incapable’ by a person of authority with whom he/she is confronted in the next clerkship.” (T5)
•	“Students are possibly more likely to ask something than would be the case with a medical lecturer.” (T1)
•	“You’re more likely to have the courage to ask questions to a student, particularly if you are afraid they might be ‘stupid’ questions.” (T2)
•	“The hierarchical gulf between tutors and tutees is clearly lower than with lecturers; this can ensure more trust and facilitate the disclosure of weaknesses or knowledge gaps and making queries or accepting help.” (T4)
	**Theme “Tutors’ closeness to being students” (5 statements)**
•	“Students are mostly closer to the subject matter – for example in the case of a clerkship. Physicians mostly delegate these tasks, meaning that practiced students are more likely to spontaneously have tips and tricks to hand and to be aware of possible obstacles within these skills – simply because they are constantly confronted with this subject matter.” (T5)
•	“For physicians, things have often already become routine, so often they don’t understand the problems of beginners.” (T1)
•	“We are more likely to be able to remember what we found difficult the first time we performed the skills and can point this out directly.” (T2)
•	“Student tutors frequently know better which points might be difficult for beginners and what can help them to master these.” (T4)
	**Theme “Competence of the tutors” (5 statements)**
•	“I see it as a disadvantage that we student tutors don’t have so much experience ourselves (so it’s both an advantage and disadvantage). There are some questions we can’t answer, where a physician would certainly know the answer. Moreover, a physician sometimes has a few ‘secret’ tips through which a skill is suddenly much easier, but you have to have already performed this skill a thousand times in order to find such tricks.” (T6)
•	We teach the skills according to standardized processes, and not how we would prefer to do it.” (T2)
•	Trained student tutors tend to teach more according to a standardized protocol than more experienced physicians, who often set subjective focuses.” (T4)
•	“A lecturer is better able to answer content-based questions which go beyond the regular content – but are equally of little relevance.” (T4)
	**Theme “Exchange and collaboration of students” (2 statements)**
•	“A preparation through tutors is useful, as one rarely gets the chance to ask students from a higher semester for their experiences and tips; particularly regarding applying for a medical clerkship, be it at home or abroad, younger students often have no idea how a clerkship proceeds, what one has to be able to do or how one should behave, and contact with older students is also useful for this.” (T2)
•	“Students train students – and this strengthens not only the collaboration among clerks in the daily routine in the clinic, but also the mutual respect.” (T5)
	**Theme “Further development of the tutors” (1 statement)**
•	“The tutors are given the possibility for personal and professional development and to assume responsibility” (T4)
	**Category 3: General aspects of the training (4 statements)**
•	“The small group situation and the format on two afternoons is also expedient, as the contents can be imparted in a structured and compact manner, without overexerting or sacrificing too much of the valuable free time in the pre-clinic”. (T4)
•	“Through student tutors, the course can be organized more cheaply and flexibly.” (T4)
•	“And moreover, such a course is a welcome change from the great amount of theoretical learning, which indeed more or less dominates our whole degree course.” (T4)
•	“As a change in the theory-laden pre-clinical everyday routine, in order to keep in view the later clinical activity.” (T4)
	**Category 4: Quality assurance and possibilities for improvement (21 statements)**
	**Theme “Skills” (9 statements)**
•	“Personally, I would remove skills like blood pressure measurement. These are things which one already performs several times a day on ward and which are also not difficult to learn. It would be good if other skills, such as surgical suture, can be further extended, so that the various suturing techniques could be consolidated.” (T5)
•	“The Schellong test is also a station that is unnecessarily long. In practice, the Schellong test is very easy to perform, as it only consists of multiple blood pressure measurement. In my view, these two skills should at least be merged (RR and Schellong).” (T2)
•	“Behavior in the operating theatre is a very important but not very well thought-out station. In my view, one would need sterile gloves to show how one puts these on (so far I have also refolded non-sterile ones to show it, but this doesn’t work so well). Moreover, I think it would be good if one had sterile surgical gowns, at least for the tutors for each group in order to show how to put these on. In terms of behavior in the operating theatre, the only thing that one can explain is all the things you’re not allowed to touch. I find it important to show how to make yourself sterile: correct washing (which we do), putting on the surgical gowns. This is precisely where I had problems my first time in the operating theatre.” (T2)
•	“Possibly combine RR-measurement and Schellong test and incorporate gastric tube as an additional skill; alternatively, maybe combine blood pressure or Schellong with Doppler examination of the vessels (ABI determination), as at these stations, frustration often arises due to redundancy.” (T4)
•	“Possibly introduce physical examination stations (heart, lungs, neurol. status), as this is only begun in the block practicum, but is hugely important for clerks, and indeed in all subjects (there, the students should perform admission examinations, and should therefore be able to perform a complete physical exam independently) – you can never begin too early with the physical exam, and never practice it enough.” (T4)
	**Theme “Organization” (8 statements)**
•	“As certificates are issued, through keeping signature lists for purposes of checking attendance, it should be ensured that the enrolled person also gets the certificate (it’s annoying but indispensable for quality assurance reasons).” (T4)
•	“Checklists for all skills should be printed out and distributed in good time and completely. For the suture course, better and more material should be procured (re-order suture in good time! Preferably procure sufficient reserves from the outset! Possibly order a suture leg from the company Fleximodell?).” (T4)
•	“The students’ enrolment one week before the start is really short notice! It’s particularly unfavorable in the lecture-free period because in this way the students cannot sufficiently plan because they can’t be certain of getting a place.” (T4)
•	“Generally, I think we should sound out more precisely how much time one needs for the individual stations. For example, one needs lots of time for ECG, permanent venous catheter and drawing blood, but very little time for behavior in the operating theatre, measuring blood pressure, injections and Schellong. Then, for example for injections, one could add a bit of theory on the flipchart to fill the time.” (T2)
•	I don’t know whether clerkship basics has already been evaluated by the students but if not, I think it should be done.” (T2)
	**Theme “Further training of the students” (4 statements)**
•	“Standardization of the tutors.” (T1)
•	“I find it important that we student tutors constantly teach other skills in the course (so rotate in some way) so that we learn all the skills better ourselves and are then better able to teach them to others.” (T6)
•	“2nd training for all. One training session is not enough to be confident and thus also radiate confidence and really explain things correctly…” (T1)
•	“Nevertheless, regular training of the students should take place so that this competence continues to be guaranteed in all medical areas.” (T5)

#### Categories

With regard to the qualitative analysis of the transcripts, 74 relevant single codes of tutors were identified. From these codes, 4 main categories were derived.

#### Definition of categories resulting from tutor quotations

Category 1: Preparation for the clerkship (30 statements).

A good preparation of the students for the clerkship through the training was central to the tutors’ statements.

•Theme “Subject-based preparation” (16 statements)

•The tutors were of the unanimous opinion that the training of practical skills in a positive, protected learning atmosphere enables better learning in the clerkship.

•Theme “Preparation for patient contact” (8 statements)

•The tutors deemed the training on the mannequin and with fellow students as an important preparation for more confidence in the patient contact. In this way, a higher quality of patient care in the clerkship can be achieved.

•Theme “Facilitation of the daily routine on ward” (6 statements)

•The tutors saw the training as an opportunity to take away students’ fears about the clerkship and to facilitate the start of the daily routine on ward.

Category 2: Leadership of the course by student tutors (19 statements)

On the whole, the tutors found the leadership of the training by students to be useful and helpful. Only the experience and knowledge of student tutors compared to experienced physicians was discussed critically.

•Theme “Fewer inhibitions towards student tutors” (6 statements)

•The tutors emphasized that a leadership of the training by student tutors helps the tutees to break down inhibitions, enabling them to ask questions, make mistakes and accept help.

•Theme “Tutors’ closeness to being students” (5 statements)

•The tutors explained that they can frequently better remember and understand beginners’ difficulties regarding skills within the clerkship than experienced physicians, and in this respect can provide good help.

•Theme “Competence of the tutors” (5 statements)

•On the one hand, the tutors revealed themselves to be critical towards their own wealth of experience and background knowledge compared to medical lecturers. One tutor pointed out that some students prefer to be taught by medical lecturers. On the other hand, they drew attention to the fact that, in contrast to experienced physicians, the tutors teach the procedures in a highly standardized manner.

•Theme “Exchange and collaboration of students” (2 statements)

•The tutorial was deemed to be useful in order to swap information and experiences regarding the degree course and clerkship in an informal manner. Moreover, the collaboration and respect among students can be strengthened.

•Theme “Further development of the tutors” (1 statement)

•The tutorial was also seen as an opportunity for the professional and personal development of the tutors and for them to assume responsibility.

Category 3: General aspects of the training (4 statements)

The tutors valued the compact format of the training in small groups as well as the flexibility and financial viability through student tutors. The practical training was seen as a positive change from the theory-laden degree course.

Category 4: Quality assurance and possibilities for improvement (21 statements)

The tutors expressed their views regarding the possibilities to improve the training with regard to contents, organizational aspects and the further training of the tutors.

•Theme “Skills” ( 9 statements)

•The tutors made concrete observations and suggestions for changing the training/skills contents. For example, they suggested merging individual skills such as the Schellong test and blood pressure measurements as these are very simple for students to learn, and redundancy may arise. Others even declared themselves in favor of completely removing blood pressure measurement or spending less time on it, due to the students’ prior experiences in the nursing practicum. As an alternative, tutors suggested, for instance, the skill of “gastric tube insertion”.

•Theme “Organization” (8 statements)

•The tutors mentioned organizational aspects such as an improvement of time management, procurement of further materials and an attendance check.

•Theme “Further training of the tutors” (4 statements)

•The tutors emphasized the importance of further, regular training for tutors as well as a standardization of the tutors. Moreover, a rotation of the tutors in the teaching of different skills was suggested in order to ensure a high qualification in all areas.

## Discussion

While still poorly studied in general, the few studies published on PAL in skills lab teaching focused on mandatory curricular skills teaching. To our knowledge, the present study is the first to investigate the acceptance of a preparatory course within the framework of PAL with regard to course evaluation from both the tutees’ and tutors’ perspective and to evaluate focused tutor training.

Evaluation by participants of the described voluntary clerkship preparation skills course was carried out from the third course onwards after two experimental implementation trials. Evaluation data gathered from 95 participants during seven course cycles turned out to be extremely positive. A comparison of pre-post self-assessment showed considerable subjective learning progress for each procedural skill taught. Even communication skills in role-playing improved significantly, although not as clearly as for the technical skills. The overall acceptance of the voluntary clerkship preparation skills course proved to be immensely high throughout all evaluation criteria. All participants were very satisfied with the course, rated the tutors’ competence as high and would recommend the course to fellow students. Preclinical students also believed that the course proved to be a helpful preparation for clinical clerkships, which indicates that the course design and contents were appropriate.

The data obtained in this study strongly suggest that a peer tutor-led skills lab course might be effective in preparing medical students for clinical clerkships and hence form the foundation for successful workplace learning. Although the general effectiveness of PAL as a teaching method has been demonstrated [6-8,12], our data do not enable the question to be answered of whether better performance in the peer tutor-led voluntary clerkship preparation skills course results in better integration and hence more effective workplace learning during clerkships. To this end, prospective trials investigating the impact on learning during clerkships would be required to confirm this hypothesis.

Furthermore, the fact that 135 students, constituting a large share of the medical faculty’s preclinical student body (19.8%), participated in the course within less than one academic year strongly emphasizes the demand for a basic skills course to prepare the students for their future practical workplace learning. Moreover, it shows that skills lab teaching enjoys a high level of acceptance even in a voluntary setting. This notion is further supported by the fact that even students who had already entered the clinical section of their degree (n = 8) decided to participate. One aspect that might account for the popularity of the clerkship preparation skills course is the appeal of a “hands-on” learning experience and the clinical perspective, even though teaching of certain basic clinical skills is already part of the mandatory preclinical curriculum. A question arising during our work with the newly established clerkship preparation skills course was whether a further differentiation of the course with the implementation of specific skills for individual medical specialties might be feasible and useful. A further matter of discussion at our own and other faculties is whether proof of basic clinical skills proficiency, e.g. by means of a course certificate, might be a suitable eligibility criterion for medical clerkships. It has been shown that blended learning experiences with virtual patients appear to make skills lab learning more effective [24]. A preparatory course for clerkship preparation might also determine whether students become more involved in clerkships and thus get more out of them.

Further support for the concept of a peer tutor-led voluntary clerkship preparation skills course is gained from evaluation data obtained from tutors. All tutors who completed the questionnaire showed a positive attitude towards the course and considered it both a helpful and necessary asset to preclinical medical education. Having gathered some experience through their own clinical clerkships, the tutors are aware of common problems encountered by students and appreciate the advantages of structured teaching. Moreover, they agree that PAL is a suitable tool for offering such standardized training, which is considered more effective than the non-standardized teaching that is often found in faculty staff-led skills training. These findings are in line with Hudson & Tonkin [7], who investigated the perception of the interaction between tutors, tutees and simulated patients during learning. All groups involved in training appreciated the concept of PAL in clinical skills training and considered that they benefited from a peer tutor-based approach. Regarding advantages and disadvantages of PAL compared to classic faculty member-led teaching, statements made by tutees and tutors were quite similar to the attitudes expressed by tutors in the current study. Tutors also approved of the training they received prior to teaching the voluntary clerkship preparation skills course. Finally, tutors considered the course to be effective in preparing preclinical students for future clerkships and interaction with patients. This attitude is obviously shared by other clinical students too, given that even third-year students applied for the clerkship preparation skills course, thus underlining the demand for such a course.

It is known that teaching quality in a skills lab and thus tutees’ learning outcome are closely linked to the tutors’ competence [25,26], making focused and structured didactic tutor training an indispensable prerequisite for PAL. Taking this into account, focused tutor training has become almost as omnipresent in medical faculties as PAL itself. However, tutor training is still mostly unstandardized and little information is available regarding suitable structures and content of tutor training, with only one publication offering first examples of a structured concept for focused tutor training [11]. In line with Heni et al., we pursued an integrative didactic approach combining medical professional content with communicative and social skills training, although the focus in the training setting described here was rather on technical procedural skills as well as on general didactic abilities, which have a high relevance for skills training such as feedback techniques and role-play. In contrast to the retrospective evaluation via online survey 6 months after the training as described by Heni et al., who also taught skills that are not primarily essential for working as a tutor, we carried out a comprehensive evaluation through questionnaires immediately after each of the three training sessions. Positive global ratings of the tutor training show that the acceptance of the didactic approach described here was high and training was perceived to be effective. Individual progress for each procedural skill was achieved by all participating tutors. Although our results gained from the evaluation of tutor training are primarily of a descriptive nature only (as is also the case in Heni et al.’s study), they are confirmed by the highly positive results of the tutors’ evaluation of the course and their teaching abilities, thus lending further support to the hypothesis that the tutor training described here is effective.

With respect to limitations, it has to be noted that the current study represents a descriptive approach, and although results obtained from the evaluation of the voluntary clerkship preparation skills course and the tutor training suggest effectiveness of the course itself and the preceding tutor training, a prospective study design is required in order to assess the ultimate impact of the clerkship preparation skills course on performance of course participants during clerkships compared to students with the same curricular education who did not participate in the course. However, such a design appears to be methodologically delicate, as students in a comparison group would be not allowed to prepare for their clerkship. Nevertheless, the immense interest in the voluntary clerkship preparation skills course and the clearly positive feedback obtained by participants show that such a course offer enjoys a high level of acceptance among medical students. The demand for practical preparation for medical clerkships appears to be high, which is consistent with tutors’ attitudes towards the course. However, the participation rate of self-selected participants from the student body was rather low, and we were only able to integrate a limited number of skills in the training. Also as stated above, subjective satisfaction with the approach pursued to achieve potential learning outcomes does not necessarily equate to effectiveness.

## Conclusions

In summary, our study demonstrates that the demand for competence-oriented clerkship preparation is high, and a peer tutor-led skills course is widely accepted and might be a feasible tool to ensure successful workplace learning. The focused didactic approach for tutor training presented here proves to be accepted and is perceived to be effective in preparing tutors for their teaching activity in this context. A prospective study design would be needed to substantiate the results objectively and confirm the effectiveness.

## Competing interests

The authors declare that they have no competing interests.

## Authors’ contributions

MB conceived of the study, participated in the design of the study, analysed the data and drafted the manuscript. MK conceived of the study, participated in the design of the study, analysed the data and drafted the manuscript together with MB. JL participated in designing the study and helped to draft the manuscript. JH helped carrying out the qualitative analysis. PW helped designing and coordinating the study and supervised the PAL programme. WH participated in designing the study and helped to draft the manuscript. JJ participated in designing the study and helped to draft the manuscript. CN conceived of the study participated in its design and helped to draft the manuscript. All authors read and approved the final manuscript.

## Pre-publication history

The pre-publication history for this paper can be accessed here:

http://www.biomedcentral.com/1472-6920/14/71/prepub
